# A Clinical Qualification Protocol Highlights Overlapping Genomic Influences and Neuro-Autonomic Mechanisms in Ehlers–Danlos and Long COVID-19 Syndromes

**DOI:** 10.3390/cimb45070379

**Published:** 2023-07-17

**Authors:** Golder N. Wilson

**Affiliations:** Department of Pediatrics, Texas Tech University Health Sciences Center, Lubbock, and KinderGenome Genetics Private Practice, 5347 W Mockingbird, Dallas, TX 75209, USA; golderwilson@gmail.com or golder.wilson@ttuhsc.edu; Tel.: +1-214-226-9869

**Keywords:** Ehlers–Danlos syndrome (EDS), post-acute COVID-19 sequelae (PACS), long COVID-19, connective tissue dysplasia, musculoskeletal disease, dysautonomia, genomic testing, collagen genes, mitochondrial DNA

## Abstract

A substantial fraction of the 15% with double-jointedness or hypermobility have the traditionally ascertained joint-skeletal, cutaneous, and cardiovascular symptoms of connective tissue dysplasia and its particular manifestation as Ehlers–Danlos syndrome (EDS). The holistic ascertainment of 120 findings in 1261 EDS patients added neuro-autonomic symptoms like headaches, muscle weakness, brain fog, chronic fatigue, dyspnea, and bowel irregularity to those of arthralgia and skin laxity, 15 of these symptoms shared with those of post-infectious SARS-CoV-2 (long COVID-19). Underlying articulo-autonomic mechanisms guided a clinical qualification protocol that qualified DNA variants in 317 genes as having diagnostic utility for EDS, six of them identical *(F2-LIFR-NLRP3-STAT1-T1CAM1-TNFRSF13B)* and eighteen similar to those modifying COVID-19 severity/EDS, including ADAMTS13/ADAMTS2-C3/C1R-IKBKG/IKBKAP-PIK3C3/PIK3R1-POLD4/POLG-TMPRSS2/TMPRSS6-WNT3/WNT10A. Also, contributing to EDS and COVID-19 severity were forty and three genes, respectively, impacting mitochondrial functions as well as parts of an overlapping gene network, or entome, that are hypothesized to mediate the cognitive–behavioral, neuro-autonomic, and immune-inflammatory alterations of connective tissue in these conditions. The further characterization of long COVID-19 natural history and genetic predisposition will be necessary before these parallels to EDS can be carefully delineated and translated into therapies.

## 1. Introduction

The study of the human being, limited by causal foibles of chance and necessity, can nevertheless take advantage of a large organism privileged by centuries of detailed observation. The biology of human systems can begin with the review of systems required for medical evaluation, a holistic approach well-complemented by a NextGen detailing of genome sequence change [1,2,3]. While the contingencies of disease pattern will never match the controlled insights from experimental study, a holistic documentation of symptoms and their translation into the pathogenetic mechanism can allow us to focus on molecular investigation. Such is the case when the full panoply of tissue laxity [4,5,6,7,8], autonomic [9,10,11], and neuromuscular [12,13] findings are ascertained in connective tissue dysplasias [8], whereby the appreciation of Ehlers–Danlos syndrome (EDS) is linked with its genetic variation to central articulo-autonomic dysplasia mechanisms [2,9,10,11] instead of peripheral phenotypes [4,5,6,7,8]. 

Evident from a systematic analysis of EDS findings [11] is a reciprocal relationship between the systems that constrain/contain body or blood [14,15] and the nervous system that coordinates their functions [16,17,18]. Disposition to tissue laxity will not only cause wear-and-tear osteoarthritis and skeletal bends from gravity (deformations like scoliosis, [11,13]) but will also provoke an adrenergic response to restore cerebral circulation deprived by vessel distensibility and lower body blood pooling [9,10,11,12,16]. Repeated adrenergic stimulation, evident even in those with minimal or benign joint hypermobility [9], produces the brain fog, stress response, and chronic fatigue of postural orthostatic tachycardia syndrome [19,20,21], the reactive allergic [11], immune [16], and inflammatory [18] symptoms of mast cell activation [21,22,23], and, through cholinergic suppression, the irregularity, reflux, and swallowing difficulties of irritable bowel syndrome [24]. 

These aspects of dysautonomia are receiving a renewed emphasis after being recognized in patients recovering from SARS-CoV-2 [25,26,27,28,29,30,31,32], an RNA beta-coronavirus producing a respiratory disease syndrome called coronavirus disease 2019 or COVID-19 [33,34,35,36,37]. The virus has caused over 650 million infections and 6.6 million deaths worldwide since its emergence from China in late 2019 [34], with complications of the acute infection extending beyond the respiratory system to involve neuromuscular [35], placental [36], and male reproductive [37] functions. Many symptoms, including particularly those of autonomic imbalance [25,26,27,28,29,30], persist for weeks or months in 6.2% of patients with documented COVID-19 infection [32]. These post-infectious symptoms have become known as a post-acute COVID-19 sequelae (PACS) or long COVID-19 syndrome that can occur after a one- to two-week course of mild, severe, or asymptomatic disease [31,32]. The frequency and timing of long COVID-19 symptoms are still being defined, and like those of EDS, are highly variable, as shown by the 59 of the 303 studies qualifying for review by Deer et al. [25].

The individual variability and heritability of certain self-reported COVID-19 symptoms [38], coupled with descriptions of X-linked COVID-19 susceptibility [39], prompted studies to define genes modulating COVID-19 susceptibility [39,40,41]. Recently reviewed studies [41] include top-down approaches analyzing interactive gene modules [42], or molecular pathways [43,44,45] altered by COVID-19 infection, and bottom-up studies focusing on individual genes using whole genome association, DNA sequencing, and CRISPR ablation analyses (see references under Table S3 of the Supplementary Materials). The overlap of symptoms between EDS [9,10,11], acute [33,34,35,36,37,38,39,40,41,42,43,44,45,46,47,48,49,50], and post-acute COVID-19 [25,26,27,28,29,30,31,32] suggested that their contributing genes might be similar, prompting analysis that could foster the application of proven therapies [6,7,8,9,10,11,19,20,21,22,24] to a novel and globally escalating disorder. 

## 2. Materials and Methods

### 2.1. Patients

The compilation of EDS patient findings profiles in Table S1 in Supplementary Materials and of the gene changes in Table S2 was performed through an observational/cross-sectional study biased by the physician- or self-referral of patients who had symptoms of EDS. EDS and developmental disability patients were seen in a private medical genetics practice from July of 2011 to August of 2017, whereby those with EDS are the sole practice focus from the latter date to October of 2020. Patients with developmental disability and/or autism had different evaluations in the private office as previously described [2]. 

Clinical evaluations and DNA testing of EDS patients were performed as described preliminarily [2,11]; the 1656 diagnosed with EDS expanded to 1899, while the 710 with systematic evaluations for the 120 findings in Supplementary Materials Table S1 to 1261, and the 727 with DNA testing to 967 (Table 1). The most recent 243 EDS patients, including 153 with DNA testing and 90 (59%) with positive results, were evaluated by telemedicine/online interaction after the private office closed in July 2018. Not meeting EDS criteria were 80 patients referred for evaluation and 64 with systematic evaluation; patients with obvious diagnoses of Marfan, Loeys–Dietz, or skeletal dysplasias were excluded from the EDS and No EDS groups. Part-time appointment at Texas Tech University Health Sciences Centers included separate genetics clinic and laboratory administrative work at that Center while coordinating the Dallas private practice.

The 967 EDS who had DNA testing were also biased toward those with insurance coverage or private means, their testing enabled by favorable out-of-pocket costs ascertained through GeneDx© company genetic counselors. Further selection bias included the systematic evaluation of all EDS patients with positive DNA results, 176 or 31% of the 568 having those evaluations at in-person follow-up visits for result counseling (all telemedicine/online patients had systematic evaluations before testing).

### 2.2. DNA Testing

Patients and/or families were given forms to consent for medical genetic evaluation/treatment and the anonymous sharing of DNA results from whole-exome sequencing (WES) during patient intake, were counseled regarding ambiguous, incomplete, or incidental/secondary findings [51], and consented to send their insurance information to the GeneDx© company for estimates of out-of-pocket costs. GeneDx genetic counselors obtained out-of-pocket cost estimates for testing, completed requisitions with generic consents for de-identified data and secondary finding sharing, and coordinated cheek swab sampling of patients and parents when available. Results using standard methods for whole-exome sequencing [52,53] with independent [54] or conjoint [55] microarray analysis were obtained by fax and/or internet portal. Results were provided with counsel by the author at follow-up clinic visits. 

### 2.3. Patient and DNA Databases

The 1979 EDS and 735 developmental disability patients having outpatient evaluations were entered into a password-protected MS Excel© GW patient database as approved by the North Texas IRB (centered at Medical City Hospital, Dallas) in 2014 (exempt protocol number 2014-054). Data on 305 EDS patients seen before 2014 were entered after approval, 68 entered as dictated by protocol guidelines after its closure on 19 December 2018 when the author closed the Medical City office. 

The 1261 EDS patients with systematic evaluations were transferred to a more comprehensive database (EDS1261GW1-23) that includes history–physical findings, specification of those related, sex, age range (2.5 years under age 10, 10 years for those over age 10.1 years), type of visit (online or clinic), referral (self, specialist, or primary physician), clinical diagnosis of hypermobile, classical, or EDS with mainly dysautonomia findings, lists of history or physical findings by category as in Table S1, and DNA testing type with indication of positive/negative results. 

The EDS1261GW1-23 database will be available upon request from the author for qualified MD or PhD researchers and will include a key for database abbreviations. Researchers can access Tables S1–S3 supplementing this article and the tables containing all DNA variants in EDS and developmental disability (manuscript in preparation—[56]), with patient numbers allowing the matching of EDS1261GW1-23 clinical data with the specific DNA variants listed in online publications. Separate listings will protect patient confidentiality but allow contact with EDS patients through the author, most of them anxious to participate in validating research.

### 2.4. Classification of Gene Products, Impacts on Tissue Elements and Processes

Genes altered in EDS patients are listed in Table S2 with numbers of variants, their identifying numbers, previously associated diseases, and functions taken from the Online Mendelian Inheritance in Man (OMIM) database at www.omim.org, accessed from June 2021 to January 2023. Genes modifying COVID-19 infection were taken from articles obtained by PubMed searches using those terms conducted throughout December 2022 and are listed with OMIM information in Table S3. Genes and their disorders are accompanied by OMIM M numbers as references to descriptive information. 

Products of the genes altered in EDS patients (Table S2) or modifying COVID-19 severity (Table S3) are classified by function (e.g., enzyme, receptor, membrane channel as shown in table legends) based on their descriptions in the OMIM entry. Classification of genes by impact on tissue element or process relies on symptoms of their associated diseases in Tables S2 and S3. These assignments are inevitably arbitrary since many associated diseases affect multiple systems and many genes are associated with more than one disease (M+ symbol). 

### 2.5. Statistics

Clinical findings were tallied from the EDS1261GW1-23 database, with gene and DNA variants tallied from the data in S2–S3, using the search, find, and sort functions in Excel. Statistical calculations involved a simple calculation of averages and standard deviations using Excel standard formulae; significant differences in these numbers at the *p* < 0.05 level were determined using online resources [57] that compared means by two-tailed t and proportions by N-1 chi-squared tests.

## 3. Results

### 3.1. EDS Patients and Their Parallel Tissue Laxity, Neurologic, and Autonomic Findings

Clinical and molecular analyses of the 1899 patients diagnosed with EDS over a 10-year period from 2011 to 2020 are summarized in Table 1, expanded from preliminary reports [2,11] as described in Methods. Table S1 shows frequencies of 80 history and 40 history findings systematically assessed on checklist forms, which included 12 history, 7 physical categories, and 28 consensus findings of EDS [4,6,58]. Although this observational data on 1261 patients are biased by referral to a medical geneticist focused on EDS, a common profile for EDS patients that transcends type or molecular change is suggested by similar finding frequencies in patients referred from orthopedic, rheumatologic, or cardiology subspecialists (manuscript in preparation—[11,56]). 

Although a detailed description of the patients evaluated for EDS along with lists of their DNA variants will be reported subsequently [56], several points bear on the comparison of Ehlers–Danlos and long COVID syndromes. First is the relevance of the findings profile in Table S1 to EDS as shown by the overall numbers of history findings averaging 34 ± 10 of 80 (36 for the 1064 females, 26 for the 197 males in Table S1) and physical findings averaging 18 ± 4.7 (19, 17 for females, males in Table S1). These numbers are significantly greater (*p* < 0.05 level) than the respective 7.2 ± 1.3 of 80 and 7.6 ± 1.3 of 40 for the 64 systematically evaluated who did not meet EDS criteria (No EDS patients). Also, supporting the EDS relevance of the genes in Table S2 are a lack of gene changes relevant to EDS in 82 patients with developmental disability, in whom some genes overlap (blue colors in Table S2) but their variants qualified as relevant to disability (manuscript in preparation, [56]).

**Figure 1:**
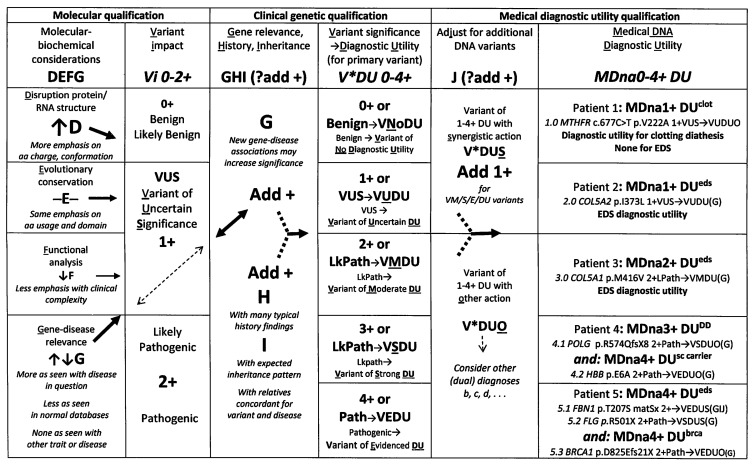
Clinical protocol for DNA variant qualification. Clinical DNA variant (column 4) and 1–4 + medical diagnostic utilities (last column) are added to consensus qualifications (column 2) as discussed in the text; DNA/protein change and gene abbreviations except for *MTHFR* (methylene tetrahydrofolate reductase) and *HBB* (beta-globin) are explained in Tables S2 and S3; single amino acid codes (A—alanine, D—aspartate, E—glutamate, I—isoleucine, L—leucine, M—methionine, P—proline, Q—glutamine, R—arginine, S—serine, T—threonine, X—stop, V—valine) used here; fs, frame-shift.

Pertinent to the clinical qualification protocol of Figure 1 are the coordinate frequencies of tissue laxity, neuromuscular, and dysautonomia findings in Table S1, which are best indicated in females, who typically have more severe symptoms due to their intrinsic hypermobility that is shown by Beighton scores [59] of 6.9 and 5.6, compared to males, who are well above the 4–5 average for the general population. More joint motion leads to wear-and-tear injuries (sprains—56% of women, ligament tears—36%, fractures—49%, stretch marks—59%, scars—43% and skeletal bends [60]) or deformities (scoliosis—25% or flat feet—46%). 

A systematic assessment is able to recognize neuromuscular symptoms like migraines or poor balance that affect a respective 60% or 61% of EDS females, with 96% of all patients having at least one of twelve neuromuscular findings by history [11]. Equally frequent occurrences due to an adrenergic response to a lower body blood pooling are the brain fog (83%) or chronic fatigue (87%) of postural orthostatic tachycardia syndrome (POTS-19, 20) in EDS females, the bowel irregularity (82%) or bloating reflux (79%) of irritable bowel syndrome (IBS-24), and the rashes (42%) or asthma dyspnea (49%) of mast cell activation syndrome (MACS, 21-22) in Table S1. It is also essential to recognize the immune [23], allergic [11], and inflammatory [21,22] abnormalities that issue from adrenergic imbalance [16], explaining the diverse nature of EDS-relevant genes and the similarity of several of them to those which modify COVID-19 severity (41, orange shading in Table S2). 

### 3.2. A Novel Clinical Protocol for DNA Variant Qualification

The novel qualification protocol in Figure 1 was developed to add biochemical and clinical considerations to the qualification of the average 12,000 DNA sequence changes found in the typical exome [61]. Sophisticated analyses by pioneering laboratories [52,53] have developed filters to separate DNA variations of potential significance for predicting or diagnosing disease from those of sufficient prevalence to qualify as benign polymorphisms. Still challenging, however, is the determination of relevant diseases, particularly when conditions like lax skin (cutis laxa) or joint muscle tenderness (fibromyalgia) may be a part of patterns like EDS that require clinical experience to recognize. 

The stepwise protocol in Figure 1, modified from prior publications [2,62], begins with either likely pathogenic consensus qualifications [63,64] or variants of uncertain significance based on the conformational grading of product disruption (step D—[65]), the evolutionary conservation of the altered gene region (step E—[66]), a functional in silico analysis (step F—[67]), and the dynamic step G of gene–disease association that increases or decreases as DNA variants are detected in similarly affected [68,69] or normal individuals [69,70,71]. 

Clinical steps are added (columns 3–5 of Figure 1) to consider abundant disease-related symptoms (step H), inheritance (step I) from relatives with these symptoms, and whether the additional or adjunct (step J) variants act by synergistic (S) or other (O) mechanisms [72]. Similar to Step G that changes as more test results associate variants with normal or diseased individuals, synergistic versus other mechanism decisions are based on prior disease associations that accumulate with reports of that variant. A DNA variant will be judged as synergistic with EDS if it is found in patients with similar connective tissue dysplasias or as relevant to other disease if found in those with unrelated conditions (see Tables S2 and S3). Each DNA variant is assigned evidenced (VEDU), strong (VSDU), moderate (VMDU) to uncertain (VUDU), or no (VNoDU) diagnostic utility (column 4, Figure 1, each patient DNA result of one or more variants then assigned 1–4 + MDna medical diagnostic utility (last column)). Adjunct synergistic or other variants are assigned V*DUS or V*DUO qualifiers, the former adding to the MDna diagnostic utility of the overall DNA result, as shown in the examples of the last column.

These examples and the advantages of molecular and clinical variant qualifications in Figure 1 will be covered in the Discussion section, within which the DNA variants in patient 4 are mentioned because they illustrate the importance of relating gene change to underlying clinical mechanisms rather than to particular disease symptoms. The mitochondrial DNA polymerase gamma (*POLG*) variants [18] would be related to the developmental disability of patient 4 (Figure 1, last column) based on that disease association (M302700+) and differently related to the dysautonomia symptoms of the 17 EDS patients in Table S2 based on the encephalopathic–gastrointestinal dysmotility symptoms of the other POLG-associated disease (M613662+). The difference applies also to the gene variants shaded blue in Table S2 which are related to EDS in patients with findings of articulo-autonomic dysplasia and to developmental disability in patients with cognitive dysfunction. The reference to the process also explains why certain Table S2 genes are shaded in green or yellow to indicate identity or similarity to those modifying COVID-19 severity, respectively, as will now be discussed. 

### 3.3. Genes Relevant to EDS

The supplemental Table S2 lists 65 genes with four or more variants in EDS patients; the 53 patients with collagen type V (COL5A1, COL5A2) gene changes were previously associated with classical EDS [6], thereby validating the relevance of these DNA testing results to EDS. The relationship of these gene changes with dysautonomia in EDS relied on the translation of the tissue laxity and autonomic findings of Table S1 into articulo-autonomic dysplasia mechanisms, including immune and inflammatory alterations from adrenergic stimulation [16]. The altered genes were classified by the nature of their products (e.g., enzymes, receptors) and by their impact on particular connective tissue or neural elements (e.g., joint, bone, general autonomic nervous system) based on their previous disease associations in column J of Table S2; abbreviations for these classifications are shown in the Table S2 legend.

**Figure 2:**
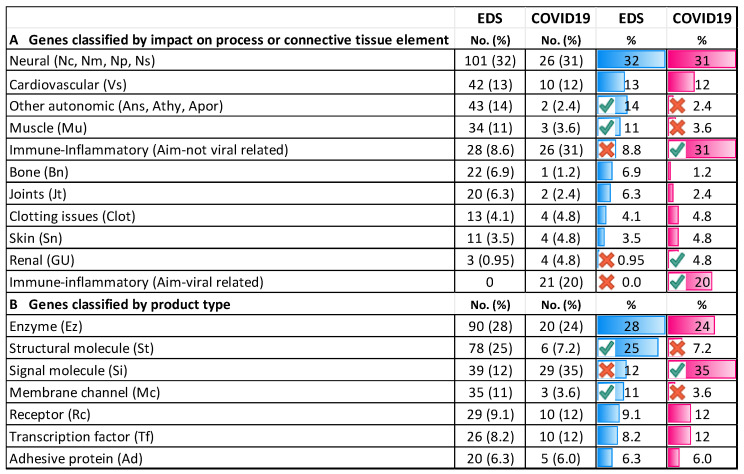
Genes relevant to EDS or COVID-19 infection by tissue element or product type. (**A**) Connective tissue element/process relations (box, Figure 2A bottom) are from associated diseases (Tables S2 and S3). COVID-19 percentages are those of 83 genes after 21 impacting viral-related processes were subtracted. (**B**) Gene product functions are explained in the legend to Table S2. COVID-19 percentages are of all 104 genes listed in Table S3 (the *PNPLA3* gene associated with gastrointestinal disease is not listed). Colors indicate relative proportions for EDS (blue) and COVID19 (red). Significantly (*p* < 0.05) lower X/ higher ↑ proportions (see Methods).

Although only 20 variant genes were related to EDS by commercial report, 317 were also related by their impact on tissue laxity and/or neuro-autonomic processes through the stepwise qualification protocol of Figure 1 (Table 1 and Table S2). While commercial reports related 181 genes to other disorders (Table 1), the relation to tissue laxity mechanisms qualified 142 altered genes as impacting tissue laxity (bone, joint, cardiovascular, clotting, muscle, skin connective tissue elements), and 101 as impacting neural and 71 autonomic functions (Figure 2A). Of particular interest regarding the comparison to the genes modifying COVID-19 severity is that 28 of the latter 71 genes associated with immune or inflammatory disorders were likely involved in the adrenergic stimulation and small fiber neuropathy processes of EDS–dysautonomia. 

Although the relations of these diverse genes to EDS will be discussed in a pending article [56], their wide genomic distribution (on every chromosome but Y from column L of Table S2) and diverse functions (column K) should be noted here. The majority encode enzymes (including those modifying collagen) or structural proteins (18 collagen genes) with regulatory products (signal, membrane channel, receptor, transcription factors) are well-represented in Figure 2B. Of great interest are the 31 of the 37 genes in the mitochondrial genome that are altered in EDS patients, which are likely acting similarly to the nuclear DNA polymerase gamma (POLG) gene changes in 17 patients to deplete the mitochondria and their energy supply to the nerve and muscle (see Section 4).

### 3.4. Genes Conferring Susceptibility to Severe COVID-19 Infection

An obvious difference between the genes contributing to EDS and those influencing the severity of COVID-19 infection [39,40,41,42,43,44] is the latter’s modulation of viral infectivity in addition to host responses. SARS-CoV-2 infection depends first on the binding of its spike (S) glycoprotein to the angiotensin-converting enzyme 2 receptor (*ACE2*, M300335), then on the cell entry of the glycoprotein complex by clathrin-mediated endocytosis and cleavage by proteases [33]. The favored route is through nasal epithelial cells with cleavage by transmembrane serine protease 2 (TMPRSS2, M602060), which, in other cells, is replaced by lysosomal cathepsin L cleavage. It is not surprising that *ACE2* and *TMPRSS2* variations in host genes are among the 104 associated with COVID-19 severity in Table S3, assembled by the literature review in January, 2023 [50], in addition to the more technical references listed below the table. 

Although 19 genes besides *ACE2* and *TMPRSS2* likely affect viral infectivity as listed in Figure 2A, relating other gene variations to COVID-19 severity requires an analysis of COVID-19 symptoms and the mechanisms producing them vis a vis the gene–disease associations in columns C and H of Table S3. The findings of acute infection, including fever, cough, fatigue, hoarse voice, loss of appetite, and delirium, occur in over 50% of patients, with diarrhea, chest pain/shortness of breath, abdominal pain, and anosmia in under 10% [33]. In 15% of patients—mostly older, male, or compromised by obesity, hypertension, diabetes, or heart disease [45]—symptoms progress to respiratory failure, renal injury, coagulation changes, and eventual multiorgan dysfunction. Long COVID-19 symptoms include prolonged versions of the above as well as many from neural, cardiovascular, autonomic, renal, and connective tissue dysfunctions that are discussed in detail below. COVID-19-related genes were classified with these acute and post-acute infectious symptoms in mind, with their associated diseases (column H, Table S3) and molecular actions (column I) implying pathogenetic mechanisms similarly for EDS in Table S2.

#### 3.4.1. Comparing COVID-19/EDS Gene Type and Distribution

The first matter to note is that the 104 COVID-19-relevant genes A are as dispersed as those of EDS except for a *CCR1/5-CXCR6* cluster at 3p22.2 (column L of Table S3). These are chemokine receptors that mediate the activation of macrophages in response to infection, wherein their associated susceptibilities to human immunodeficiency, hepatitis, and West Nile viruses suggest that they impact virus-related aspects of the immune/inflammatory response. These genes are included with the 21 that affect viral response (Aim-V or blue print in columns D and J of Table S3), with the vaccine-targeted ACE2 receptor belonging to this group, but here is related to kidney function since it is expressed in kidney, like its homologue ACE1. 

The 26 genes judged to be more related to cellular autonomic–immune response mechanisms (Aim) include *IFNAR1/2* or *STAT2* that regulate interferon action and *TNFRSF13B1* that participates in T-cell signaling; all three genes are associated with immunodeficiencies in Table S3. Other genes intimately involved in the host response to viral infection include the *ICAM1* and *IFITM3* genes that encode cell adhesion molecules. The latter include the interferon alpha-1 *IFNA1* gene associated with Epstein–Barr virus susceptibility and the IRF9 binding component of the interferon-induced transcription factor that forms a complex with the mentioned *STAT2* gene product. Many of these gene products will likely become targets for vaccines like those targeting the SARS-CoV-2 spike protein [33]. 

**Figure 3:**
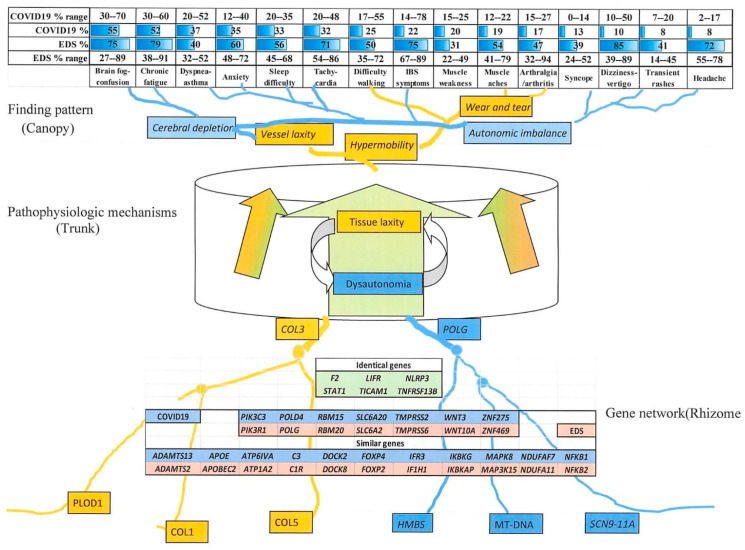
Genes and symptoms related to EDS and COVID-19. Genes related to EDS (Table S2) and COVID-19 infection (Table S3) are envisioned as overlapping parts of a network (rhizome below) connected through pathogenic mechanisms (trunk sap, phloem) to common symptoms of EDS (Table S1) and long COVID-19 (canopy above). EDS symptom ranges are for females over age 10.5 years from the EDS1261database; long COVID percentages and ranges are taken from Figure 2 of the work by Deer et al. [37].

In addition to the expected genes modulating viral infectivity or host resistance are many associated with systemic diseases, including 13, with an impact on embryonic development, that are given the Dev classification in column F of Table S3. These include the *LIFR* gene that is associated with the multiple anomalies of the Stuve–Wiedemann syndrome (M601559) and the *WNT3* gene associated with limb agenesis (M273395). Another 26 are associated with diseases having connective tissue dysplasia symptoms (red print in Table S3 column J), including particularly the *NLRP3* gene that, like *LIFR,* is relevant to both EDS and COVID-19 severity (Figure 3). Its associated disease (M191900) has joint pain, muscle aches, and symptoms of mast cell activation (M191900), as well as a tissue laxity–dysautonomia theme echoed by the *ATP6V1A* gene-associated lax skin disease (M617403) that has tall stature, aortic dilation, and joint contractures. Another seven associations in Table S3 have molecular similarities to EDS-relevant disorders (red print underlined), the *FURIN* (M136950, [73,74]) and *TEAD3* (M603170, [75]) genes impacting transforming growth factor beta pathways (as do the *FBN1* and *TGFB/R* genes altered in EDS patients), the *DPP7* (M610537) and *DPP9* (M608258) peptidases that cleave proline residues abundant in collagens [76], the *DDR1* receptor (M600408) that binds fibrillar collagens [15], the lung surfactant protein *SFTPD* (M178635) that has collagen-like glycine–hydroxyproline–hydroxylysine residues, and the *NDUFAF79* gene (M615898) involved in the assembly of mitochondrial complex I (recall the 31 EDS patients with *MT-ND* component gene alterations in Table S2).

Especially of interest, based on the renal complications of COVID-19, are eight genes associated with renal disease (purple print in Table S3 column J), four of them impacting vessels that include *ACE1*, *AGT*, *AGTR1* related to angiotensinogen–angiotensin I/II conversions. The *ACE2* gene encoding the SARS-CoV-2 nasal epithelial receptor is included in this group, a metalloproteinase that is also expressed in the vascular endothelium of heart and kidney, but not yet associated with a hereditary disease despite its X chromosome location. The *ADAMTS13*-encoded metalloprotease is similar to the EDS-related ADAMTS2 product in Figure 3, associated with a clotting diathesis and renal disease (M274150). The latter symptoms and those of other disorders associated with these kidney-related genes are reminiscent of the thrombotic complications of COVID-19 [44]. Three genes in EDS patients affect the kidney (Table S2): the sodium chloride co-transporter *SLC12A3* associated with Gitelman syndrome M263800, uromodulin *UMOD* associated with renal tubular disease M263800, and *PKD1* associated with polycystic kidney disease M173100. Based on the latter disease’s aneurysms and their thrombotic complications, genes affecting kidney function are classified as having vascular impact (Vs) in Tables S2 and S3.

Ten genes are associated with neurologic disorders (green print in Table S3 column J), including the *IRF3* gene associated with an encephalopathy (M616532) conferring headaches and brain fog and the *RAB7A* gene with a form of Charcot–Marie–Tooth disease (M600882) that is associated with 16 EDS-relevant genes. The apolipoprotein E protein is associated with Alzheimer (M104310+) and heart disease (M617347), the NPC1 cholesterol trafficking regulator responsible for CNS lipid accumulation in Niemann–Pick disease (M257250). These have similar actions to the *LPIN1* (M605518) and *LDR* (M606945) gene variants in EDS patients (Table S2). 

The X chromosome androgen receptor AR that, like many EDS-contributing genes, impacts muscle (M313200+) and joins *ACE2*, *TLR7,* and six other X chromosome genes as potential factors in male susceptibility to COVID-19. The latter contrasts with the 85% female preponderance in EDS (Table 1), although sex ratios in long COVID-19 may be more equal (see below).

The percentage column of Figure 2A excludes the 21 genes judged to be more related to viral entry/proliferation (virulence) and calculates the gene class proportions relative to the 82 genes involved in host mechanisms. Genes related to immune/inflammatory processes to viral infection (Aim) still dominate, comprising 26 of 82, or 31%, of those influencing COVID-19 severity versus 8.8% of the 317 contributing to EDS from Table S2. The proportions of genes impacting other elements or processes related to EDS versus COVID-19 in Figure 2A are similar for cardiovascular (42 or 13% versus 10 or 13%), neural (101 or 32% versus 26 or 31%), clotting (4.1 versus 4.8%), and skin (3.5 versus 4.8%). They differ significantly (red or green circles) in categories of other autonomic (14 versus 2.4%) or muscle (11 versus 3.2%) and substantially for bone (22 versus 1,2%), joints (6.3 versus 2.4%), and renal (0.95 versus 4.8%).

Gene product types may reflect the importance of structural proteins in EDS and immune signaling after COVID-19 infection, with 76 or 25% of EDS-relevant genes being structural (St), versus 6 or 7.2% for COVID-relevant genes, and 39 or 12% of the former having signal (Si) functions versus 20 or 35% of the latter in Figure 2B. Other product proportions are similar, with 3 (11%) of 82 COVID-19-related genes having mitochondrial connections, including *STAT2* (elongated mitochondria in muscle), *TLR3* (mitochondrial antiviral pathway), and *NDUFAF7* (assembly of mitochondrial complex I). Each of these has similarities to EDS-relevant genes, as shown in Figure 3, with 13% of the overall 40 mitochondrially related genes being similar to the 317 related to EDS. 

#### 3.4.2. Comparing Individual Symptoms and Genes Relevant to COVID-19 with Those of Eds

As stated in the Introduction, a pattern of persisting symptoms dominated by fatigue, brain fog, breathing problems, and joint muscle pain became apparent in patients recovering from SARS-CoV-2 [25,26,27,28,29,30,31]. This finding constellation became known as post-acute COVID-19 sequalae (PACS) or long COVID-19. A recent report estimated that 6.2% of people had one of three symptom clusters (persistent fatigue with bodily pain or mood swings, cognitive problems, ongoing respiratory problems) after COVID-19 infection [32]. 

The variable periods, vacillating intensity, and subjective nature of long COVID-19 symptoms have been difficult to characterize, but a unifying theme is autonomic dysfunction, as demonstrated by measures of orthostatic intolerance and postural orthostatic tachycardia syndrome [26,27,28,29,30]. The systematic review by Deer et al. from 2021 [37] adopted standard phenotypic descriptions for symptoms [77] and included 59 articles among 303 that looked at clinical manifestations 3 weeks or more after initial symptoms of COVID-19 infection (outpatients) or hospital discharge (inpatients). 

As with the EDS patients described here, the 81 cohorts reviewed [37] were heterogenous with a various mix of post-infectious timing, outpatient–hospital–intensive care, a physical examination self-report, sex, and age (overall male-to-female ratio of 1.2 to 1 estimated from their data). Also, the variable frequencies of laboratory/pathology findings were similar—some suggestive of long-term organ damage after COVID-19—and the inevitable ambiguity of symptom descriptions pointed out by Deer et al. [25]: how chronic was the fatigue; was it steatosis or fatty liver? Although standard nomenclature for symptoms [77] is an asset, it does not group symptoms by clinical mechanism. 

#### 3.4.3. Comparison of EDS and Long COVID-19 Symptoms

Symptoms common to EDS and long COVID are shown at the top of Figure 3 that depicts the relations of findings and genes in EDS by analogy to Tolkien’s Ents. A tissue laxity–dysautonomia entome is imagined with a converging network of contributing genes at the bottom (roots) and a diverging network of symptoms at the top (branching canopy), with the two connected through pathophysiologic mechanisms like articulo-autonomic dysplasia (flowing channels of phloem or sap in the trunk). Peripheral genes with less impact on the central mechanism will have less disruptive variations in affected patients, while those like *COL3A1* (M120180) will act as nodes in these gene networks and cause more numerous and severe symptoms. 

The large percentage ranges for symptoms in both patient groups reflects the heterogeneity of patient ascertainment (clinic, online, retrospective in EDS, different hospitalized outpatient cohorts, post-infection times for COVID-19) and the subjective nature of reported findings. All symptoms, ordered by percentage in COVID-19 patients, are more frequent in EDS, although ranges are slightly more compatible. Symptoms of autonomic imbalance (brain fog, chronic fatigue, asthma dyspnea, sleep difficulties, and tachycardia) are common in both EDS and long COVID-19 (Figure 3), asthma is a consequence of mast cell activation [21,22], the other four of the postural orthostatic tachycardia syndrome [19,20,27].

Less common in long COVID-19 than EDS are IBS symptoms, and those of orthostatic hypotension, like syncope and dizziness (Figure 3). Neurological symptoms like difficulty walking and poor balance, muscle weakness, myalgia, and frequent headaches occur in both, as does joint pain that is common in EDS, autoimmune illnesses, or other prolonged infections like mononucleosis. Occurring occasionally but not chronic in EDS are the cough (16%), chest pain (14%), congestion (10%), sore throat (4%), and low-grade fever (4%) reported by Deer et al. [25], which are symptoms possibly related to a persisting viral infection. 

#### 3.4.4. Similar Genes Relevant to EDS and COVID-19 Severity

Genes highlighted by variance or expression in both disorders include the *F2* prothrombin gene (M176930) related to bleeding disorders and the metalloproteases *ADAMTS2* (M6045539) and *ADAMTS13* (M604134), with the latter gene product interacting with the von Willebrand factor that had 18 coding variants in EDS (Table S2). Ratios of the ADAMTS13 and VWF proteins are related to thrombosis and COVID-19 mortality [43,44,52,53], recalling the 13 genes and 34 variants in EDS patients that impact clotting functions, including 15 patients with *VWF* gene variants (Table S2). 

The shared *LIFR* leukemia inhibitory factor receptor (M151443) with immunoglobulin/cytokine domains and the *NLRP3* (M606416) pyrin-like genes could be involved in the inflammatory response to COVID-19 as well as the enhanced inflammation from adrenergic stimulation in EDS and other conditions [10,11,18,78]. Similar dualities for the *STAT1* (M600555), *TNFRSF13B* (M6049097), and *TICAM1* (M607601) genes may apply since the first two are associated with immunodeficiency disorders and the last confers susceptibility to encephalopathy from herpes virus infection (Table S3). 

Among the 18 similar genes are complement components *C3* (M120700) and *C1R* (613785) that, with the *NFKB1* (M164011)-*NFKB2* (M164012), *IFR3* (M603734)-*IF1H1* (M606951) genes (Figure 3) and others, could also mediate inflammatory and autoimmune symptoms. The *POLG* (M174763) and *NDUFA11* (M612638) gene variants in EDS are most easily related to neurologic and autonomic symptoms, with a high certainty for the similar *POLD4* (M611525) gene (Table S3) and the *NDUFAF7* (M615898) gene likely presenting a neurologic impact [41]. The *PIK3C3* (M602609) gene similarity to *PIK3R1* (M171833) that is associated with tissue laxity is an example of 28 COVID-19-related genes having molecular or symptom similarities to connective tissue dysplasias (red print in Table S3 column J).

## 4. Discussion

This clinical genetic study of EDS relates its quantified finding pattern to underlying articulo-autonomic dysplasia mechanisms and the multiple gene variants found by NextGen DNA sequencing. Major results are the connected tissue laxity–neural symptoms of EDS, their relation to disparate nuclear and mitochondrial genes, and the potential similarities of these relationships to those of acute or long COVID-19. The study illustrates the potential strengths and limitations of genomic analysis as summarized below. 

### 4.1. Clinical–DNA Correlation in EDS

The core of this paper is the molecular and clinical qualification protocol of Figure 1 that grades gene relevance to a pathogenic mechanism rather than to particular disease findings. Relevance to mechanism is judged by the associations of gene alterations with disease, a dynamic qualification that may increase or decrease as additional genomic analyses associate DNA variants with disease or normalcy. For EDS patients, the mechanisms are the reciprocal ones of tissue laxity and dysautonomia (articulo-autonomic dysplasia), as inferred from the association of patient DNA variation (Table S2) with those findings (Table S1). For long COVID-19, putative gene changes have been associated with a severity of the acute disease, whose mechanisms are interpreted by matching acute symptoms with those of the associated diseases in Table S3. Symptom and gene similarities shown in Figure 2 and Figure 3 suggest that long COVID-19 severity will correlate with genes modifying the acute form of disease, a hypothesis that remains to be tested. A similarity of gene products and mechanisms between EDS- and COVID-19-relevant genes in Figure 2 suggests that similar gene changes and mechanisms operate in these two syndromes, fostering trials of proven EDS–dysautonomia therapies in patients with acute and post-infectious COVID-19.

The complete ascertainment of patient findings and their relation to pathogenetic mechanisms is necessary for understanding genetic influence on diseases like EDS as shown by the molecular and medical qualification protocol of Figure 1. Clinical [2] as well as molecular genetic [63,64,65,66,67] interpretation of DNA variants is required to overcome the medical distrust of genomic results by: (1) minimizing the use of the unhelpful “variant of uncertain significance” for DNA qualification; (2) adding connotations for less helpful variants (VUDU, VnoDU) or those suggestive of dual diagnoses (V*DUO); and (3) emphasizing that DNA changes may support but never make a “molecular” diagnosis [72] without an experienced clinical correlation.

DNA variants become candidates for disease correlation in the way their genes used to become candidates for marker loci before all genes could be sequenced. The correlation of gene action with pathogenic mechanisms deduced from symptom pattern then “elects” or rejects candidate gene relevance to disease as more patients have genomic (gene unbiased) analysis. Although in vitro functional analyses demonstrating mutational disruption are considered requisites for relating DNA change to disease, the need to replicate patient genetic background and pertinent tissue designs sets them as subject for future research rather than as an immediate aid to variant interpretation.

The protocol in Figure 1 replaces the false yes/no dichotomy of molecular diagnosis with 0–4 + degrees of diagnostic utility for each DNA variant and ultimately of 0–4 + medical DNA (MDna) diagnostic utility for each patient’s variant combination. This graded and multivariate approach is particularly important for complex traits like hypermobility, dysautonomia, or EDS; their severity is a network property of multiple genes. While pivotal genes like *COL3* [7] or *CHST14* (M608429)] can act as nodes in gene networks to produce extreme phenotypes like the vascular or musculocontractural (M601776) types of EDS, their spectral consequences from different mutations mandate medical DNA diagnoses such as those for homozygous sickle cell mutations in Jamaica [79].

#### 4.1.1. Examples of Clinical Qualification

By returning to the last column of Figure 1, we can emphasize the importance of clinical qualification by experienced physicians through several examples:

Patient 1 has an *MTHFR* 677C>T variant that is present at a 30% frequency in normal databases [68], its purported disease association (clotting diathesis) a multifactorial condition regulated by many genes and physiologic factors. The variant is therefore qualified as one of uncertain significance for another disorder (VUDUO) with a 1 + medical diagnostic utility for clotting diathesis. The fact that no DNA variants with diagnostic utility for EDS was found does not contradict the clinical diagnosis of a multifactorial disorder. 

Patients 2 and 3 had single variants numbered 2.0 and 3.0 in the collagen type V alpha chain genes that are frequently altered in EDS patients [6]. Both genes were qualified as having relevance to EDS (G); the conservative isoleucine-to-leucine substitution of variant 2.0 was given a 1+ for variant impact and a subsequent uncertain diagnostic utility (VUDU). The more disruptive methionine-to-valine change of variant 3.0 g is given a 2+ and VMDU qualification in Figure 1. There were no additional pluses for substantial EDS history findings (H), for concordant findings and variant presence in relatives (I), or adjunct variants with synergistic action (J). The stepwise qualification resulted in respective 1+ and 2 + medical diagnostic utility scores.

Patient 4 with developmental disability (DD) had two variants, firstly, 4.1 in the *POLG* gene [18] that can cause significant encephalopathy in some patients, but gastrointestinal–neuromuscular symptoms like those of EDS in others (M613662—see Tables S2 and S3). The *POLG* gene, among 20 that were variant in DD and EDS patients (bottom of Table S2), was qualified as relevant to the patient’s disability in this case. The additional beta-globin gene variant 4.2 was qualified with evident diagnostic utility for another diagnosis (VEDUO), that of sickle cell carrier status [79]. It is expected that DNA variations like the *HBB* gene sickle mutation with long histories of association will automatically receive evident diagnostic utilities, yet a correlation with patient clinical–laboratory findings is still required before that utility is interpreted as a clinical diagnosis for the patient. No DNA finding should be registered as a molecular diagnosis [72], except for those with an evident utility for the laboratory that supports a clinical diagnosis with an integration of all clinical–molecular findings by a physician [2].

Patient 5 had three variants, with 5.1 judged to have a significant impact (DEF) in the collagen type III gene (M120270) that is well-correlated with EDS (G), 5.2 an equally impactful termination mutation in the profilaggrin gene (*FLG*, M135940), and 5.3 judged to have utility for another diagnosis, that of breast/ovarian cancer predisposition. The 5.1 *COL3A1* gene variant was inherited from a mother with symptoms (matSx), adding a point for inheritance (I) to that for typical history (H). The qualification of the 5.2 *FLG* variant as having synergistic action (+ for J) based on its 40 variations in EDS patients (Table S2) emphasizes the advantages of a protocol that qualifies by symptom pattern and pathogenic mechanisms like those of skin inflammation with a decreased tissue constraint in EDS [80]. Less informative was the commercial laboratory approach that associates *FLG* variants with the single symptom of scaly skin (ichthyosis vulgaris, M145700). Molecular and clinical qualification appropriately assigns a 4+ diagnostic utility to the *COL3A1*-*FLG* variant combination, with the symptom pattern favoring hypermobile EDS even though frequent associations of *COL3A1* variants with vascular EDS (M130050) might add screening for aneurysms to its usual management.

#### 4.1.2. Advantages of Molecular and Clinical Qualification

The additional clinical correlation emphasizes the entire profile of disease (i.e., all skin/skeletal, neuromuscular, and dysautonomia findings of an EDS patient) rather than a single one like kyphoscoliosis [81], an approach essential for relating syndrome pattern to mechanism. Thus, mitochondrial DNA polymerase gamma (*POLG*) variants [18] would be related to the developmental disability of patient 4 (Figure 1, last column) based on that disease association (M302700+), but rather to dysautonomia symptoms of the 17 EDS patients in Table S2 based on the encephalopathic–gastrointestinal dysmotility symptoms of its other associated disease (M613662+). 

Changing “molecular diagnosis” [72] to diagnostic utility, minimizing the unhelpful “variant of uncertain significance qualification” [63], and adding qualifiers with connotations like VUDU, VnoDU, and V*DUO (dual) in Figure 1 would lessen physician skepticism about genomic analysis [82]. It would also make clear that even the most established molecular change (e.g., homozygosity for the HBB p.Glu6Ala variant of patient 4 in Figure 1) may not diagnosis sickle cell anemia in Jamaican patients with mild symptoms [79]. 

This large patient collection barely sketches the genomics of EDS and shows the massive numbers of appropriately qualified DNA testing results that will be needed to provide the understanding, diagnosis, and informed management of multifactorial disease. The need for a clinical correlation of DNA variants is underlined by several genetic properties reviewed in this study. Not only are different connective tissue dysplasia phenotypes produced by different types or locations of mutations in the same gene (Tables S2 and S3), but many exon-fabricated genes like collagens [14,15] will have shared domains that could be mutated to provide similar phenotypes. The latter can also result from mutations in different collagen genes since several types of collagens participate in fibril assembly [76]. These considerations make one gene-one type/disease matches [6,7] unlikely, and molecular diagnoses without clinical correlation [72] untenable for EDS and, by extrapolation, for any genetically influenced disease like COVID-19. Collaborative interpretations of variant diagnostic utility—disease relevance by molecular geneticists and appropriate physician subspecialists per Figure 1 protocol—are required if DNA testing is to become a prime contributor to precision medicine. 

### 4.2. Distribution and Nature of EDS and COVID-19-Related Genes

The genes associated with EDS (317) or COVID-19 severity (104) are distributed on all chromosomes (except for Y and 8, 13, 16, 20 for COVID-19) with clusters at 2q32.2 (*COL5A2/COL3*), 3p24.1 (*SCN5/10/11A*), 11q23.3 (*SCN2/4B*), and 21q22.3 (*COL6A1/A2*) for EDS and at 3p22.2 (*CCR1/5-CXCR6*) for COVID-19 (Tables S2 and S3, column L). Genes impacting mitochondrial function include 31 of 37 in mitochondrial DNA and nuclear genes encoding mitochondrial proteins, including ten EDS-related (*NDUFA*-*11*/*S3*, *OPA1*, *TYMP*, etc.) and three COVID-19-related (*STAT2, TLR3, NDUFAF7*) genes. The encoding of products with structural (*SURF1*, *MT-trRNA*), respiratory enzyme component (*MT-ND*/*CO*), adhesive (*NUBPL*), or DNA polymerase (*POLG*) functions by these genes suggests an influence on EDS by the depletion of mitochondrial number and/or energy coupling. Mitochondrial roles in aging [83] and immunity/inflammation [84,85,86] may explain the influence on COVID-19 infection.

The diversity of function and location (Tables S2 and S3, Figure 2) of genes influencing EDS or COVID-19 are consistent with their participation in networks regulating connective tissue integrity and its reciprocal autonomic regulation. Both functions would be impacted by gene variation in EDS, while autonomic imbalance with its immune and inflammatory dysregulation would be more impacted in COVID-19. A primordial structural operon might be imagined for initial metazoan transitions, the duplication and realignment of protein domains shown by the binding of acetylcholinesterase to collagen by COLQ protein (M603033), the interspersion of VWF motifs in COL3 [15] and COL7 [14] proteins, and the services of abundant collagen type I as anchor for immune molecules [] as well as core for other types during fibril formation [76]. This modular pleiotropy is supported by the 184 (62%) of the 298 associated disorders with at least three tissue dysplasia symptoms in Table S2 (orange shading). 

The attribution of variant genes in EDS to tissue element or process (Figure 2A) fostered a comparison to the 104 genes relevant to COVID-19 severity (Table S3), with 18 genes similar and 6 identical between the two groups (Figure 3). These include variant genes with parallel impacts or functions like *ADAMTS2/F2/PIK3R1* influencing EDS; *ADAMTS13/F2/PIK3C3* influencing COVID-19 that impact clotting tissue laxity; *LIFR/NLRP3/STAT1/T1CAM1/TNFRSF13B* (both) plus *C1R/IF1H1/NFKB2* (EDS) and *C3/IFR3/NFKB1* (COVID-19) that impact immunity-inflammation; *SLC6A2* (EDS) and *SLC6A20* (COVID-19) that have transport functions; and *POLG/FOXP2/RBM20/WNT10A/ZNF469* (EDS) and *POLD4/FOXP4/RBM15/WNT3/ZNF275* (COVID-19) that have DNA polymerase/regulatory functions (Figure 3, Tables S2 and S3). The occurrence of small-fiber neuropathy [12,56] and thyroid dysfunction (Table S1) in EDS (Table S1) and COVID-19 [59], along with the many shared joint muscle and dysautonomia symptoms in Figure 3, support the operation of overlapping gene networks in these disorders. 

### 4.3. Connections of Signs and Symptoms to Genes as “Entomes”

These networks may be analogized to Tolkien’s Ents, their genes as rhizomes, clinical mechanisms as trunks, and medical problems as the diverging branches of the entome (Figure 3). Clinical findings caused by these overlapping gene networks will have the oppositely branching, widely shared traits like whole-body pain, muscle weakness, and adrenaline surges (stems) being more frequent in EDS or long COVID-19 than their component symptoms of arthralgia, myalgia, headaches, poor balance, or chronic fatigue (findings as small branches, canopy, upper part of Figure 3). 

Entomes differ from gene modules or molecular pathways by connecting genes to sign and symptom patterns: genes converge to and symptoms derive from central pathogenic mechanisms. Key genes and common symptoms are nodes of their respective networks (lower part of Figure 3). The idea of an entome connects these mirroring networks of genes and symptoms through pathogenetic mechanisms, their divergent clinical findings like the distributed flood debris that can be related to normal structures only by the knowledge of floodwater force and direction. 

### 4.4. Implications for Future Research

#### 4.4.1. Expanded Studies on EDS and Long COVID-19 Symptoms and Outcomes

The systematic ascertainment of patient findings as listed in Table S1 for EDS can be applied to those with long COVID-19, including objective measures like echocardiography–vascular screening [7], tilt table [19], nerve conduction [12], intestinal motility [24], or mast cell mediators [21]. The inclusion of these assessments in evolving evaluation protocols for patients with long COVID-19 [31] could provide objective descriptors [77] and outcome measures [87] as functions of time and gene variation, respectively. A prototype would be the outcome measures for COVID-19 infection in patients with rheumatic diseases [88]. Anecdotal reports from EDS patients suggest that they are more likely to suffer prolonged symptoms of COVID-19, another relationship to be tested using physiologic and genetic measures.

When these more complete protocols were used for patients with EDS and COVID-19 infection, their objective clinical profiles could be compared to those of other infectious conditions like multisystem inflammatory syndrome or Kawasaki disease in children [89]. COVID-19 hospitalization and mortality rates were not increased in patients with fibromyalgia [46], but this symptomatic and heterogenous diagnosis ignores many findings of EDS–dysautonomia and has limited association with biomarkers [90]. 

#### 4.4.2. Future Therapies for EDS, COVID-19, and the Related Symptoms of Aging

A further study of musculoskeletal and mitochondrial dysfunction [91,92,93] in EDS and acute/long COVID-19 could justify the trials of promising dietary [19], physical therapy [94], and exercise [95] protocols in both disorders. Important objectives regarding long COVID-19 are to associate symptom frequencies and outcome measures with defined post-infection time periods, then determine whether the genes influencing acute COVID-19 severity (Table S3) also influence the duration and disability of its post-infectious phases.

Given the similarity of many EDS symptoms like skin laxity or poor balance to those of the old [11] and the elderly who are more vulnerable to COVID-19 [45], will the 108 genes that impact connective tissue elements in Figure 2 (EDS), the 28 associated with connective tissue dysfunction in Table S3 (COVID-19), and the implications of mitochondrial dysfunction [83] in both disorders indicate kindred mechanisms in aging? If so, then unified therapy approaches could be applied to the flexible [4], frail [45], or infected [33] that could, as basic science distills cause from the present correlations, involve autologous transplants with variant-edited [96] stem cells [91,97].

## 5. Conclusions

1. The prevalence of dysautonomia findings is equal to those of tissue laxity in 1261 EDS patients with systematic evaluation, with their underlying articulo-autonomic dysplasia mechanisms being essential for comparisons to long COVID-19.
2. Comparison of EDS to COVID-19 included 15 overlapping dysautonomia–musculoskeletal symptoms in those with persisting symptoms and 24 identical or similar genes among the 84 moderating the severity of acute infection.
3. These many gene changes are hypothesized to act through a network or entome to produce overlapping Ehlers–Danlos or long COVID-19 syndrome profiles, their disease symptoms (canopy), genes (rhizome), and connecting pathogenetic mechanisms (trunk-phloem) visualized as entomes by analogy to Tolkien’s Ents.
4. If a better characterization of the natural history and genetic predispositions to long COVID-19 validates the common musculoskeletal, neuro-autonomic, and immune/inflammatory mechanisms hypothesized in this article, then EDS-proven exercise, dietary, and medication therapies may be tried in the 6.2% of COVID-19 patients who endure persisting symptoms.

## Figures and Tables

**Table 1:** Ehlers–Danlos syndrome patients and their DNA testing.

Patient Group	All EDS	Female	Male	No EDS
Total number (No.) T	1899	1553	346	80
Number with systematic evaluations (%T)	1261 (66)	1064 (69)	197 (57)	64 (80)
“ with DNA testing (%T)	967 (51)	816 (53)	151 (44)	23 (29)
“ with potentially significant DNA variant (V)	568	480	88	4
“ with variant relevant to EDS/*DD* by Co. (%V)	20 (3.5)	16 (3.3)	4 (4.5)	0
“ with variant relevant to other Dx by Co. (%V)	181 (32)	154 (32)	27 (31)	0
“ with variant relevant to EDS/*DD* by Au. (%V)	566 (99)	478 (99)	88 (100)	4 (100)
“ with primary MT DNA variant (%V)	93 (16)	79 (16)	14 (16)	0
“ with primary nuclear to MT DNA variant (%V)	19 (3.4)	18 (3.8)	1 (1.1)	0

The All EDS column shows all of the EDS patients’ meeting criteria in the upper row and those having systematic evaluation for the findings in Supplemental Materials Table S1 in the next. More females (1553 or 82%) than males were referred and more had DNA testing (816 or 84%), with the 80 patients not meeting criteria (58, No EDS) having more standard evaluations (80%) but less DNA testing (23 of 80 or 29%). EDS patients had few DNA variants (20 of 568 or 3.5%) qualified as relevant to EDS by the testing companies (Co., mainly GeneDx, Gaithersburg, MD, USA), most of them qualified as of uncertain significance or pathogenic for other diagnoses (181 or 32%). Of the 967 having DNA testing, 568 EDS patients had variants or variant combinations qualified as potentially significant, and 566 were judged relevant to EDS by the protocol in Figure 1. Positive DNA results included 93 patients with at least one variant in mitochondrial DNA and 19 patients with at least one variant in a nuclear gene like POLG [18] that targeted its protein product to mitochondria.

## Data Availability

All DNA data will be offered to the ClinGen and Mitomap databases after journal publication, 60–65% of DNA variants already entered as will be shown in the list of all EDS-related DNA variants in a subsequent publication [56]. It is hoped that publication after peer review will support the association of these DNA variants with EDS as interpreted by the author. The databases of EDS and developmental disability DNA variants will be available in the Supplementary Materials of the pending publication [56]. The deidentified Excel database of 1261 patients with interval ages, sex, findings, and positive/negative DNA testing results without variant details (EDS1261GW 1-23) will be available upon request to the author.

## Supplementary Materials

The following supporting information can be downloaded at: https://www.mdpi.com/article/10.3390/cimb45070379/s1, Supplementary Materials consist of an Excel file (Supplementary Materials for EDS–COVID-19 gene network.xls) that contains three supplemental Tables S1–S3. N EDS1261GW1-23 database containing de-identified patient findings is available upon request to the author; qualified MD or PhD researchers are able to match patients in the database with supplemental DNA data in this and a pending publication [56]. References [33,34,38,39,41,42,74,75,98,99,100,101,102,103,104,105,106,107,108,109,110,111,112,113,114,115,116,117,118,119] are cited in the supplementary materials.

## References

[1] Weerakkody R.A., Vandrovcova J., Kanonidou C., Mueller M., Gampawar P., Ibrahim Y., Norsworthy P., Biggs J., Abdullah A., Ross D. (2016). Targeted next-generation sequencing makes new molecular diagnoses and expands genotype–phenotype relationship in Ehlers–Danlos syndrome. Genet Med..

[2] Wilson G.N. (2019). Genomic Analysis of 727 Patients with Ehlers-Danlos Syndrome I: Clinical Perspective Relates 23 Genes to a Maternally Influenced Arthritis-Adrenaline Disorder. J. Biosci. Med..

[3] Junkiert-Czarnecka A., Pilarska-Deltow M., Bąk A., Heise M., Latos-Bieleńska A., Zaremba J., Bartoszewska-Kubiak A., Haus O. (2022). Next-Generation Sequencing of Connective Tissue Genes in Patients with Classical Ehlers-Danlos Syndrome. Curr. Issues Mol. Biol..

[4] Tinkle B.T., Levy H.P. (2019). Symptomatic joint hypermobility: The hypermobile type of Ehlers-Danlos syndrome and the hypermobility spec-trum disorders. Med. Clin. North Am..

[5] Malfait F., Francomano C., Byers P., Belmont J., Berglund B., Black J., Bloom L., Bowen J.M., Brady A.F., Burrows N.P. (2017). The 2017 international classification of the Ehlers-Danlos syndromes. Am. J. Med. Genet. C Semin. Med. Genet..

[6] Bowen J.M., Sobey G.J., Burrows N.P., Colombi M., Lavallee M.E., Malfait F., Francomano C.A. (2017). Ehlers-Danlos syndrome, classical type. Am. J. Med. Genet. C Semin. Med. Genet..

[7] Byers P.H., Belmont J., Black J., De Backer J., Frank M., Jeunemaitre X., Johnson D., Pepin M., Robert L., Sanders L. (2017). Diagnosis, natural history, and management in vascular Ehlers-Danlos syndrome. Am. J. Med. Genet. C Semin. Med. Genet..

[8] McKusick V.A. (1955). Heritable disorders of connective tissue: I. The clinical behavior of hereditary syndromes. J. Chronic Dis..

[9] Gazit Y., Nahir A.M., Grahame R., Jacob G. (2003). Dysautonomia in the joint hypermobility syndrome. Am. J. Med..

[10] De Wandele I., Rombaut L., Leybaert L., Van de Borne P., De Backer T., Malfait F., De Paepe A., Calders P. (2014). Dysautonomia and its underlying mechanisms in the hypermobility type of Ehlers–Danlos syndrome. Semin. Arthr. Rheum..

[11] Wilson G.N. (2019). Clinical Analysis Supports Articulo-Autonomic Dysplasia as a Unifying Pathogenic Mechanism in Ehlers-Danlos Syndrome and Related Conditions. J. Biosci. Med..

[12] Cazzato D., Castori M., Lombardi R., Caravello F., Bella E.D., Petrucci A., Grammatico P., Dordoni C., Colombi M., Lauria G. (2016). Small fiber neuropathy is a common feature of Ehlers-Danlos syndromes. Neurology.

[13] Henderson F.C., Austin C., Benzel E., Bolognese P., Ellenbogen R., Francomano C.A., Ireton C., Klinge P., Koby M., Long D. (2017). Neurological and spinal manifestations of the Ehlers-Danlos syndromes. Am. J. Med. Genet. C Semin. Med. Genet..

[14] Wegener H., Leineweber S., Seeger K. (2013). The vWFA2 domain of type VII collagen is responsible for collagen binding. Biochem. Biophys. Res. Commun..

[15] Lisman T., Raynal N., Groeneveld D., Maddox B., Peachey A.R., Huizinga E.G., de Groot P.G., Farndale R.W. (2006). A single high-affinity binding site for von Willebrand factor in collagen III, identified using synthetic triple-helical peptides. Blood.

[16] Lorton D., Bellinger D.L. (2015). Molecular Mechanisms Underlying β-Adrenergic Receptor-Mediated Cross-Talk between Sympathetic Neurons and Immune Cells. Int. J. Mol. Sci..

[17] Wilson G.N., Tonk V.S. (2020). Mitochondrial Dysfunction Contributes to Ehlers-Danlos Syndrome—A Patient Presentation. J. Biol. Life Sci..

[18] Gaudó P., Emperador S., Garrido-Pérez N., Ruiz-Pesini E., Yubero D., García-Cazorla A., Artuch R., Montoya J., Bayona-Bafaluy M.P. (2020). Infectious stress triggers a POLG-related mitochondrial disease. Neurogenetics.

[19] Vernino S., Bourne K.M., Stiles L.E., Grubb B.P., Fedorowski A., Stewart J.M., Arnold A.C., Pace L.A., Axelsson J., Boris J.R. (2021). Postural orthostatic tachycardia syndrome (POTS): State of the science and clinical care from a 2019 National Institutes of Health Expert Consensus Meeting—1. Auton. Neurosci..

[20] Benarroch E.E. (2012). Postural Tachycardia Syndrome: A Heterogeneous and Multifactorial Disorder. Mayo Clin. Proc..

[21] Wang E., Ganti T., Vaou E., Hohler A. (2021). The relationship between mast cell activation syndrome, postural tachycardia syndrome, and Ehlers-Danlos syndrome. Allergy Asthma Proc..

[22] Monaco A., Choi D., Uzun S., Maitland A., Riley B. (2022). Association of mast-cell-related conditions with hypermobile syn-dromes: A review of the literature. Immunol. Res..

[23] Zhang S.Z., Wang Q.Q., Yang Q.Q., Gu H.Y., Yin Y.Q., Li Y.D., Hou J.-C., Chen R., Sun Q.-Q., Sun Y.-F. (2019). NG2 glia regulate brain innate immunity via TGF-β2/TGFBR2 axis. BMC Med..

[24] Thwaites P.A., Gibson P.R., Burgell R.E. (2022). Hypermobile Ehlers–Danlos syndrome and disorders of the gastrointestinal tract: What the gastroenterologist needs to know. J. Gastroenterol. Hepatol..

[25] Deer R.R., Rock M.A., Vasilevsky N., Carmody L., Rando H., Anzalone A.J., Basson M.D., Bennett T.D., Bergquist T., Boudreau E.A. (2021). Characterizing Long COVID: Deep Phenotype of a Complex Condition. Ebiomedicine.

[26] Dani M., Dirksen A., Taraborrelli P., Torocastro M., Panagopoulos D., Sutton R., Lim P.B. (2020). Autonomic dysfunction in ‘long COVID’: Rationale, physiology and management strategies. Clin. Med..

[27] Raj S.R., Arnold A.C., Barboi A., Claydon V.E., Limberg J.K., Lucci V.M., Numan M., Peltier A., Snapper H., Vernino S. (2021). Long-COVID postural tachycardia syndrome: An American Autonomic Society statement. Clin. Auton. Res..

[28] Ceban F., Ling S., Lui L.M.W., Lee Y., Gill H., Teopiz K.M., Rodrigues N.B., Subramaniapillai M., Di Vincenzo J.D., Cao B. (2022). Fatigue and cognitive impairment in Post-COVID-19 Syndrome: A systematic review and meta-analysis. Brain Behav. Immun..

[29] Dotan A., David P., Arnheim D., Shoenfeld Y. (2022). The autonomic aspects of the post-COVID19 syndrome. Autoimmun. Rev..

[30] Batiha G.E., Al-Kuraishy H.M., Al-Gareeb A.I., Welson N.N. (2022). Pathophysiology of Post-COVID syndromes: A new perspective. Virol. J..

[31] Thaweethai T., Jolley S.E., Karlson E.W., Levitan E.B., Levy B., McComsey G.A., McCorkell L., Nadkarni G.N., Parthasarathy S., Singh U. (2023). Recover Consortium. Development of a Definition of Postacute Sequelae of SARS-CoV-2 Infection. JAMA.

[32] Wulf Hanson S., Abbafati C., Aerts J.G., Al-Aly Z., Ashbaugh C., Ballouz T., Blyuss O., Bobkova P., Bonsel G., Global Burden of Disease Long COVID Collaborators (2022). Estimated Global Proportions of Individuals with Persistent Fatigue, Cognitive, and Respiratory Symptom Clusters following Symptomatic COVID-19 in 2020 and 2021. JAMA.

[33] Guo G., Ye L., Pan K., Chen Y., Xing D., Yan K., Chen Z., Ding N., Li W., Huang H. (2020). New Insights of Emerging SARS-CoV-2: Epidemiology, Etiology, Clinical Features, Clinical Treatment, and Prevention. Front. Cell Dev. Biol..

[34] World Health Organization. https://covid19.who.int.

[35] Dale L. (2022). Neurological Complications of COVID-19: A Review of the Literature. Cureus.

[36] Tossetta G., Fantone S., Delli Muti N., Balercia G., Ciavattini A., Giannubilo S.R., Marzioni D. (2022). Preeclampsia and severe acute respiratory syndrome coronavirus 2 infection: A systematic review. J. Hypertens..

[37] Delli Muti N., Finocchi F., Tossetta G., Salvio G., Cutini M., Marzioni D., Balercia G. (2022). Could SARS-CoV-2 infection affect male fertility and sexuality?. Apmis.

[38] Williams F.M.K., Freidin M.B., Mangino M., Couvreur S., Visconti A., Bowyer R.C.E., Le Roy C.I., Falchi M., Mompeó O., Sudre C. (2020). Self-Reported Symptoms of COVID-19, Including Symptoms Most Predictive of SARS-CoV-2 Infection, Are Heritable. Twin Res. Hum. Genet..

[39] Asano T., Boisson B., Onodi F., Matuozzo D., Moncada-Velez M., Maglorius Renkilaraj M.R.L., Zhang P., Meertens L., Bolze A., Materna M. (2021). X-linked recessive TLR7 deficiency in ~1% of men under 60 years old with life-threatening COVID-19. Sci. Immunol..

[40] Ishaq U., Malik A., Malik J., Mehmood A., Qureshi A., Laique T., Zaidi S.M.J., Javaid M., Rana A.S. (2021). Association of ABO blood group with COVID-19 severity, acute phase reactants and mortality. PLoS ONE.

[41] Dieter C., Brondani L.A., Leitão C.B., Gerchman F., Lemos N.E., Crispim D. (2022). Genetic polymorphisms associated with susceptibility to COVID-19 disease and severity: A systematic review and meta-analysis. PLoS ONE.

[42] Sharma P., Pandey A.K., Bhattacharyya D.K. (2021). Determining crucial genes associated with COVID-19 based on COPD Findings. Comput. Biol. Med..

[43] Philippe A., Gendron N., Bory O., Beauvais A., Mirault T., Planquette B., Sanchez O., Diehl J.-L., Chocron R., Smadja D.M. (2021). Von Willebrand factor collagen-binding capacity predicts in-hospital mortality in COVID-19 patients: Insight from VWF/ADAMTS13 ratio imbalance. Angiogenesis.

[44] Favaloro E.J., Henry B.M., Lippi G. (2021). Increased VWF and Decreased ADAMTS-13 in COVID-19: Creating a Milieu for (Micro)Thrombosis. Semin. Thromb. Hemost..

[45] Lynch S.M., Guo G., Gibson D.S., Bjourson A.J., Rai T.S. (2021). Role of Senescence and Aging in SARS-CoV-2 Infection and COVID-19 Disease. Cells.

[46] Amital M., Ben-Shabat N., Amital H., Buskila D., Cohen A.D., Amital D. (2021). COVID-19 associated hospitalization in 571 patients with fibromyalgia—A population-based study. PLoS ONE.

[47] Barros A., Queiruga-Piñeiro J., Lozano-Sanroma J., Alcalde I., Gallar J., Fernández-Vega Cueto L., Alfonso J.F., Quirós L.M., Merayo-Lloves J. (2022). Small fiber neuropathy in the cornea of Covid-19 patients associated with the generation of ocular surface disease. Ocul. Surf..

[48] McFarland A.J., Yousuf M.S., Shiers S., Price T.J. (2021). Neurobiology of SARS-CoV-2 interactions with the peripheral nervous system: Implications for COVID-19 and pain. PAIN Rep..

[49] Díaz-Alberola I., Espuch-Oliver A., García-Aznar J.M., Ganoza-Gallardo C., Aguilera-Franco M., Sampedro A., Jiménez P., López-Nevot M. (2022). Common Variable Immunodeficiency Associated with a De Novo IKZF1 Variant and a Low Humoral Immune Response to the SARS-CoV-2 Vaccine. J. Clin. Med..

[50] Tutal E., Ozaras R., Leblebicioglu H. (2022). Systematic review of COVID-19 and autoimmune thyroiditis. Travel Med. Infect. Dis..

[51] Green R.C., Berg J.S., Grody W.W., Kalia S.S., Korf B.R., Martin C.L., McGuire A.L., Nussbaum R.L., O’Daniel J.M., Ormond K.E. (2013). American College of Medical Genetics and Genomics. ACMG recommendations for reporting of incidental findings in clinical exome and genome sequencing. Genet. Med..

[52] Bamshad M.J., Ng S.B., Bigham A.W., Tabor H.K., Emond M.J., Nickerson D.A., Shendure J. (2011). Exome sequencing as a tool for Mendelian disease gene discovery. Nat. Rev. Genet..

[53] Yang Y., Muzny D.M., Reid J.G., Bainbridge M.N., Willis A., Ward P.A., Braxton A., Beuten J., Xia F., Niu Z. (2013). Clinical Whole-Exome Sequencing for the Diagnosis of Mendelian Disorders. N. Engl. J. Med..

[54] Wyandt H.E., Wilson G.N., Tonk V.S. (2017). Microarray analysis performed by a variety of commercial laboratories for patients with developmental disability and autism, including the author-associated laboratory at Texas Tech University that used standard methods and interpretation as outlined. Chromosome Structure and Variation: Heteromorphism, Polymorphism, and Pathogenesis.

[55] Retterer K., Scuffins J., Schmidt D., Lewis R., Pineda-Alvarez D., Stafford A., Schmidt L., Warren S., Gibellini F., Kondakova A. (2015). Assessing copy number from exome sequencing and exome array CGH based on CNV spectrum in a large clinical cohort. Genet Med..

[56] Wilson G.N. (2023). A network of genes (entome) is responsible for congruent tissue laxity and dysautonomia findings in hypermobility spectrum/Ehlers-Danlos syndrome disorders.

[57] MedCalc Software Ltd. https://www.medcalc.org/calc.

[58] Ehlers-Danlos Society (2017). Criteria for EDS types. https://www.ehlers-danlos.com/2017-eds-international-classification/.

[59] The Ehlers-Danlos Society Beighton Maneuvers Illustrated. https://www.ehlers-danlos.com/assessing-joint-hypermobility/.

[60] Yonko E.A., LoTurco H.M., Carter E.M., Raggio C.L. (2021). Orthopedic considerations and surgical outcomes in Ehlers–Danlos syndromes. Am. J. Med. Genet. C Semin. Med. Genet..

[61] Ng P.C., Levy S., Huang J., Stockwell T.B., Walenz B.P., Li K., Axelrod N., Busam D.A., Strausberg R.L., Venter J.C. (2008). Genetic Variation in an Individual Human Exome. PLOS Genet..

[62] Wilson G.N., Tonk V.S. A protocol for qualifying DNA variants associated with complex diseases like Ehlers-Danlos syndrome.

[63] Richards S., Aziz N., Bale S., Bick D., Das S., Gastier-Foster J., Grody W.W., Hegde M., Lyon E., Spector E. (2015). ACMG Laboratory Quality Assurance Committee. Standards and guidelines for the interpretation of sequence variants: A joint consensus recommendation of the American College of Medical Genetics and Genomics and the Association for Molecular Pathology. Genet. Med..

[64] MacArthur D.G., Manolio T.A., Dimmock D.P., Rehm H.L., Shendure J., Abecasis G.R., Adams D.R., Altman R.B., Antonarakis S.E., Ashley E.A. (2014). Guidelines for investigating causality of sequence variants in human disease. Nature.

[65] Tam B., Sinha S., Wang S.M. (2020). Combining Ramachandran plot and molecular dynamics simulation for structural-based variant classification: Using TP53 variants as model. Comput. Struct. Biotechnol. J..

[66] Genome Browser, University of California Santa Clara. https://genome.ucsc.edu/.

[67] Hu Z., Yu C., Furutsuki M., Andreoletti G., Ly M., Hoskins R., Adhikari A.N., Brenner S.E. (2019). VIPdb, a genetic Variant Impact Predictor Database. Hum. Mutat..

[68] ClinVar. https://www.ncbi.nlm.nih.gov/clinvar/.

[69] (2019). MITOMAP: A Human Mitochondrial Genome Database. http://www.mitomap.org.

[70] Lek M., Karczewski K.J., Minikel E.V., Samocha K.E., Banks E., Fennell T., O’Donnell-Luria A.H., Ware J.S., Hill A.J., Cummings B.B. (2016). Exome Aggregation Consortium. Analysis of protein-coding genetic variation in 60,706 humans. Nature.

[71] Gnomad. https://gnomad.broadinstitute.org/.

[72] Posey J.E., Harel T., Liu P., Rosenfeld J.A., James R.A., Coban Akdemir Z.H., Walkiewicz M., Bi W., Xiao R., Ding Y. (2017). Resolution of Disease Phenotypes Resulting from Multilocus Genomic Variation. N. Engl. J. Med..

[73] Noda K., Kitagawa K., Miki T., Horiguchi M., Akama T.O., Taniguchi T., Taniguchi H., Takahashi K., Ogra Y., Mecham R.P. (2020). A matricellular protein fibulin-4 is essential for the activation of lysyl oxidase. Sci. Adv..

[74] Torre-Fuentes L., Matías-Guiu J., Hernández-Lorenzo L., Montero-Escribano P., Pytel V., Porta-Etessam J., Gómez-Pinedo U., Matías-Guiu J.A. (2021). ACE2, TMPRSS2, and Furin variants and SARS-CoV-2 infection in Madrid, Spain. J. Med. Virol..

[75] Pairo-Castineira E., Clohisey S., Klaric L., Bretherick A.D., Rawlik K., Pasko D., Walker S., Parkinson N., Fourman M.H., Russell C.D. (2021). Genetic mechanisms of critical illness in COVID-19. Nature.

[76] Weis M.A., Hudson D.M., Kim L., Scott M., Wu J.J., Eyre D.R. (2010). Location of 3-Hydroxyproline Residues in Collagen Types I, II, III, and V/XI Implies a Role in Fibril Supramolecular Assembly. J. Biol. Chem..

[77] Human Phenotype Ontology. https://hpo.jax.org/app/.

[78] Wilson G.N., Tonk V.S. (2020). Demon Genes May Deform Common Syndromes: Collagen VI Gene Change in Down Syndrome Unifies the Medical and Molecular Approach to Hypermobility Disorders. J. Biosci. Med..

[79] Serjeant G.R., Vichinsky E. (2018). Variability of homozygous sickle cell disease: The role of alpha and beta globin chain variation and other factors. Blood Cells, Mol. Dis..

[80] Woźniak E., Owczarczyk-Saczonek A., Lange M., Czarny J., Wygonowska E., Placek W., Nedoszytko B. (2023). The Role of Mast Cells in the Induction and Maintenance of Inflammation in Selected Skin Diseases. Int. J. Mol. Sci..

[81] Abdalla E.M., Rohrbach M., Bürer C., Kraenzlin M., El-Tayeby H., Elbelbesy M.F., Nabil A., Giunta C. (2015). Kyphoscoliotic type of Ehlers-Danlos Syndrome (EDS VIA) in six Egyptian patients presenting with a homogeneous clinical phenotype. Eur. J. Pediatr..

[82] Hunter D.J., Drazen J.M. (2019). Has the Genome Granted Our Wish Yet?. N. Engl. J. Med..

[83] Jang J.Y., Blum A., Liu J., Finkel T. (2018). The role of mitochondria in aging. J. Clin. Investig..

[84] Shenoy S. (2020). Coronavirus (Covid-19) sepsis: Revisiting mitochondrial dysfunction in pathogenesis, aging, inflammation, and mortality. Inflamm. Res..

[85] Xie J.H., Li Y.Y., Jin J. (2020). The essential functions of mitochondrial dynamics in immune cells. Cell. Mol. Immunol..

[86] Field C.S., Baixauli F., Kyle R.L., Puleston D.J., Cameron A.M., Sanin D.E., Hippen K.L., Loschi M., Thangavelu G., Corrado M. (2020). Mitochondrial Integrity Regulated by Lipid Metabolism Is a Cell-Intrinsic Checkpoint for Treg Suppressive Function. Cell Metab..

[87] Weinfurt K.P., Reeve B.B. (2022). Patient-Reported Outcome Measures in Clinical Research. JAMA.

[88] Talarico R., Aguilera S., Alexander T., Amoura Z., Antunes A.M., Arnaud L., Avcin T., Beretta L., Bombardieri S., Burmester G.R. (2021). The impact of COVID-19 on rare and complex connective tissue diseases: The experience of ERN ReCONNET. Nat. Rev. Rheumatol..

[89] Wilson G.N., Tonk V.S. (2020). Do severe complications like aneurysms from Kawasaki and Kawasaki-like infections arise in children with underlying articulo-autonomic dysplasia/Ehlers-Danlos syndrome?. Ann. Ped. Res..

[90] D’agnelli S., Arendt-Nielsen L., Gerra M.C., Zatorri K., Boggiani L., Baciarello M., Bignami E. (2018). Fibromyalgia: Genetics and epigenetics insights may provide the basis for the development of diagnostic biomarkers. Mol. Pain.

[91] Yue F., Era T., Yamaguchi T., Kosho T. (2023). Pathophysiological Investigation of Skeletal Deformities of Musculocontractural Ehlers–Danlos Syndrome Using Induced Pluripotent Stem Cells. Genes.

[92] Lewis M.T., Levitsky Y., Bazil J.N., Wiseman R.W. (2022). Measuring Mitochondrial Function: From Organelle to Organism. Methods Mol Biol..

[93] Grünewald A., Voges L., Rakovic A., Kasten M., Vandebona H., Hemmelmann C., Lohmann K., Orolicki S., Ramirez A., Schapira A.H.V. (2010). Mutant Parkin Impairs Mitochondrial Function and Morphology in Human Fibroblasts. PLoS ONE.

[94] Russek L.N., Block N.P., Byrne E., Chalela S., Chan C., Comerford M., Frost N., Hennessey S., McCarthy A., Nicholson L.L. (2023). Presentation and physical therapy management of upper cervical instability in patients with symptomatic generalized joint hypermobility: International expert consensus recommendations. Front. Med..

[95] Mitchell T., Barlow C.E. (2011). Review of the Role of Exercise in Improving Quality of Life in Healthy Individuals and in Those with Chronic Diseases. Curr. Sports Med. Rep..

[96] Doudna J.A. (2020). The promise and challenge of therapeutic genome editing. Nature.

[97] Nancarrow-Lei R., Mafi P., Mafi R., Khan W. (2017). A Systemic Review of Adult Mesenchymal Stem Cell Sources and their Multilineage Differentiation Potential Relevant to Musculoskeletal Tissue Repair and Regeneration. Curr. Stem Cell Res. Ther..

[98] van Der Made C.I., Simons A., Schuurs-Hoeijmakers J., van den Heuvel G., Mantere T., Kersten S., van Deuren R.C., Steehouwer M., van Reijmersdal S.V., Jaeger M. (2020). Presence of genetic variants among young men with severe COVID-19. JAMA.

[99] Delorey T.M., Ziegler C.G., Heimberg G., Normand R., Yang Y., Segerstolpe Å., Abbondanza D., Fleming S.J., Subramanian A., Montoro D.T. (2021). COVID-19 tissue atlases reveal SARS-CoV-2 pathology and cellular targets. Nature.

[100] Namkoong H., Edahiro R., Takano T., Nishihara H., Shirai Y., Sonehara K., Tanaka H., Azekawa S., Mikami Y., Lee H. (2022). DOCK2 is involved in the host genetics and biology of severe COVID-19. Nature.

[101] COVID-19 Host Genetics Initiative (2021). Mapping the human genetic architecture of COVID-19. Nature.

[102] Chu J., Xing C., Du Y., Duan T., Liu S., Zhang P., Cheng C., Henley J., Liu X., Qian C. (2021). Pharmacological inhibition of fatty acid synthesis blocks SARS-CoV-2 replication. Nat. Metab..

[103] Feng S., Song F., Guo W., Tan J., Zhang X., Qiao F., Guo J., Zhang L., Jia X. (2022). Potential genes associated with COVID-19 and comorbidity. Int. J. Med. Sci..

[104] Baldassarri M., Picchiotti N., Fava F., Fallerini C., Benetti E., Daga S., Valentino F., Doddato G., Furini S., Giliberti A. (2021). Shorter androgen receptor polyQ alleles protect against life-threatening COVID19 disease in European males. EBioMedicine.

[105] Daniloski Z., Jordan T.X., Wessels H.H., Hoagland D.A., Kasela S., Legut M., Maniatis S., Mimitou E.P., Lu L., Geller E. (2021). Identification of required host factors for SARS-CoV-2 infection in human cells. Cell.

[106] Boussier J., Yatim N., Marchal A., Hadjadj J., Charbit B., El Sissy C., Carlier N., Pène F., Mouthon L., Tharaux P.L. (2022). Severe COVID-19 is associated with hyperactivation of the alternative complement pathway. J. Allergy Clin. Immunol..

[107] Nakanishi T., Pigazzini S., Degenhardt F., Cordioli M., Butler-Laporte G., Maya-Miles D., Bujanda L., Bouysran Y., Niemi M.E., Palom A. (2021). Age-dependent impact of the major common genetic risk factor for COVID-19 on severity and mortality. J. Clin. Investig..

[108] Niemi M.E.K., Daly M.J., Ganna A. (2022). The human genetic epidemiology of COVID-19. Nat. Rev. Genet..

[109] Bastard P., Rosen L.B., Zhang Q., Michailidis E., Hoffmann H.H., Zhang Y., Dorgham K., Philippot Q., Rosain J., Béziat V. (2020). Autoantibodies against type I IFNs in patients with life-threatening COVID-19. Science.

[110] Zhang Q., Bastard P., Liu Z., Le Pen J., Moncada-Velez M., Chen J., Ogishi M., Sabli I.K., Hodeib S., Korol C. (2020). Inborn errors of type I IFN immunity in patients with life-threatening COVID-19. Science.

[111] Saponi-Cortes J.M., Rivas M.D., Calle-Alonso F., Sanchez J.F., Costo A., Martin C., Zamorano J. (2021). IFNL4 genetic variant can predispose to COVID-19. Sci. Rep..

[112] Wu L., Zhu J., Liu D., Sun Y., Wu C. (2021). An integrative multiomics analysis identifies putative causal genes for COVID-19 severity. Genet. Med..

[113] Wu P., Ding L., Li X., Liu S., Cheng F., He Q., Xiao M., Wu P., Hou H., Jiang M. (2021). Trans-ethnic genome-wide association study of severe COVID-19. Commun. Biol..

[114] Patrick M.T., Zhang H., Wasikowski R., Prens E.P., Weidinger S., Gudjonsson J.E., Elder J.T., He K., Tsoi L.C. (2021). Associations between COVID-19 and skin conditions identified through epidemiology and genomic studies. J. Allergy Clin. Immunol..

[115] Meng Y., Zhang Q., Wang K., Zhang X., Yang R., Bi K., Chen W., Diao H. (2021). RBM15-mediated N6-methyladenosine modification affects COVID-19 severity by regulating the expression of multitarget genes. Cell Death Dis..

[116] Grimaudo S., Amodio E., Pipitone R.M., Maida C.M., Pizzo S., Prestileo T., Tramuto F., Sardina D., Vitale F., Casuccio A. (2021). PNPLA3 and TLL-1 polymorphisms as potential predictors of disease severity in patients with COVID-19. Front. Cell. Dev. Biol.

[117] Latini A., Agolini E., Novelli A., Borgiani P., Giannini R., Gravina P., Smarrazzo A., Dauri M., Andreoni M., Rogliani P. (2020). COVID-19 and genetic variants of protein involved in the SARS-CoV-2 entry into the host cells. Genes.

[118] Wang F., Huang S., Gao R., Zhou Y., Lai C., Li Z., Xian W., Qian X., Li Z., Huang Y. (2020). Initial whole-genome sequencing and analysis of the host genetic contribution to COVID-19 severity and susceptibility. Cell Discov..

[119] Biering S.B., Sarnik S.A., Wang E., Zengel J.R., Leist S.R., Schäfer A., Sathyan V., Hawkins P., Okuda K., Tau C. (2022). Genome-wide bidirectional CRISPR screens identify mucins as host factors modulating SARS-CoV-2 infection. Nat. Genet..

